# Cancer risk among residents of Rhineland-Palatinate winegrowing communities: a cancer-registry based ecological study

**DOI:** 10.1186/1745-6673-3-12

**Published:** 2008-06-06

**Authors:** Andreas Seidler, Gaël Paul Hammer, Gabriele Husmann, Jochem König, Anne Krtschil, Irene Schmidtmann, Maria Blettner

**Affiliations:** 1 Federal Institute for Occupational Safety and Health (BAuA), Berlin, Germany; 2 Institute of Medical Biostatistics, Epidemiology and Informatics (IMBEI), Johannes Gutenberg-University Mainz, Germany; 3 Cancer Registry of Rhineland-Palatinate, Notification Office, Mainz, Germany; 4 Cancer Registry of Rhineland-Palatinate, Registration Office, Mainz, Germany

## Abstract

**Aim:**

To investigate the cancer risk among residents of Rhineland-Palatinate winegrowing communities in an ecological study.

**Methods:**

On the basis of the Rhineland-Palatinate cancer-registry, we calculated age-adjusted incidence rate ratios for communities with a medium area under wine cultivation (>5 to 20 percent) and a large area under wine cultivation (>20 percent) in comparison with communities with a small area under wine cultivation (>0 to 5 percent). In a side analysis, standardized cancer incidence ratios (SIR) were computed separately for winegrowing communities with small, medium and large area under wine cultivation using estimated German incidence rates as reference.

**Results:**

A statistically significant positive association with the extent of viniculture can be observed for non-melanoma skin cancer in both males and females, and additionally for prostate cancer, bladder cancer, and non-Hodgkin lymphoma in males, but not in females. Lung cancer risk is significantly reduced in communities with a large area under cultivation. In the side-analysis, elevated SIR for endocrine-related tumors of the breast, testis, prostate, and endometrium were observed.

**Conclusion:**

This study points to a potentially increased risk of skin cancer, bladder cancer, and endocrine-mediated tumors in Rhineland-Palatinate winegrowing communities. However, due to the explorative ecologic study design and the problem of multiple testing, these findings are not conclusve for a causal relationship.

## Introduction

Some previous studies point to a potential association between pesticide exposure resp. farming or winegrowing and lymphoma [1-5] or multiple myeloma [6-11], brain cancer [12-14], prostate cancer [15], or bladder cancer [16,17]. However, the mechanisms of the suspected carcinogenic effects of pesticides are widely unclear.

Among the hypothesis on potential carcinogenic mechanisms from pesticides, the endocrine mediated effects have received much attention. Several pesticides interact with endocrine receptors in vitro or have endocrine-mediated effects in laboratory animals in vivo: The European Union has listed over 40 pesticides suspected to interfere with the hormone system of humans and wildlife [18]. As endocrine-related mechanisms play an etiologic role in several cancers in humans, the potential association between exposure to pesticides with endocrine activity and cancer incidence has been discussed in the last years. Many epidemiological studies have, for example, examined the relationship between pesticides and breast cancer [19]. However, although endogenous and exogenous estrogens are known to play a causal role in the aetiology of breast cancer, the to date epidemiological and experimental evidence is not conclusive for an association between exposure to organochlorine pesticides and breast cancer incidence (for an overview, see [19]). According to Barlow [19], the evidence on other endocrine-related tumour sites (testes, prostate, endometrium) is too sparse to draw any conclusions concerning pesticides.

Rhineland-Palatinate is the federal state with the most extensive winegrowing in Germany: About 3 percent of the Rhineland-Palatinate area is under wine cultivation. Therefore, a potential pesticide exposure of the residential population might be assumed. Actual deposit measurements in one Rhineland-Palatinate wine district (Moselle region) point to an ongoing insecticide (parathione) and herbicide (atrazine, simazine) exposure of the residential population [20]. Repeatedly, a suspected increase in cancer incidence has been a subject of concern in the mentioned region. The aim of the present ecological study is therefore to investigate the cancer risk among residents of Rhineland-Palatinate winegrowing communities compared to the cancer risk among residents of communities with a small area under wine cultivation.

## Materials and methods

### Study population and study area

Each Rhineland-Palatinate winegrowing community (n = 503, out of 2,305 communities in Rhineland-Palatinate) was categorized according to the proportion of area under wine cultivation of the whole community area (small: >0 to 5 percent; medium: >5 to 20 percent; large: >20 percent area under wine cultivation; see Table 1) based on official data for 1996. 1.3 percent of the total area of communities with a small area under cultivation is area under wine, respectively, 12.5 percent of the total area of communities with a medium area under cultivation, and 31.4 percent of the total area of communities with a large area under cultivation. Table 1 gives some characteristics of the Rhineland-Palatinate study region.

**Table 1:** Characteristics of the Rhineland-Palatinate vineyard area

	Rhineland-Palatinate*	Area under wine (% of community area)		
	Total	> 0%, ≤ 5%	>5%, ≤ 20%	>20%
Communities	2,305	162	171	170
Total area (ha)	1,984,688	222,736	200,709	129,444
Area under wine (ha)	69,043	2,996	25,101	40,683
% area under wine	3.5%	1.3%	12.5%	31.4%
Inhabitants (per ha)	4,000,567 (2.02)	564,210 (2.53)	526,486 (2.62)	301,193 (2.33)
Inhabitants per community (median, min-max)	566 (6–184,752)	1,188 (72–99,750)	1,193 (95–80,535)	984 (84–40,110)

* All data pertain to Dec 31st, 1996 (Statistisches Landesamt Rheinland-Pfalz 2006)

### Cancer registry data

This study is based on cancer cases registered in the Rhineland-Palatinate cancer registry which covers a population of approximately 4,000,000 persons. We included all malignant tumours plus benign brain and CNS tumours and brain and CNS tumours of uncertain behaviour. Furthermore, we included malignant bladder tumours plus carcinoma in situ and tumours of uncertain behaviour of the bladder. Since January 2000 all Rhineland-Palatinate physicians and dentists are legally obliged to report incident cancer cases to the cancer registry. Therefore, all above mentioned cancers diagnosed between 2000 and 2003 and reported until mid-2005 were included. The following items are registered: diagnosis (ICD-10); topography and morphology (ICD-O-2); staging (TNM); incidence date; most valid basis of diagnosis; occasion of first detection; initial treatment; last occupation and longest held occupation; and date and cause of death (where appropriate). Population figures and data on area under wine cultivation were obtained from the statistical office of Rhineland-Palatinate.

### Statistical methods

Completeness of the Rhineland-Palatinate cancer registry varies with time, region, physician's specialization and type of cancer. This had to be considered in our analysis. Completeness is estimated by the ratio of reported cases to estimated cases for Rhineland-Palatinate calculated from a national pooling of cancer registry data [21,22]. In communities with a small area under wine cultivation, the completeness (excluding non-melanotic skin cancer) is about 80 percent in males and 79 percent in females. Concerning lymphohaematopoetic malignancies, the completeness is considerably lower; in communities with a small area under cultivation, only 62 percent of Non-Hodgkin lymphoma (NHL) in males and 64 percent in females are reported to the registry.

### Primary "internal" analysis of incidence ratio ratios for communities with a medium or large area under cultivation in comparison with communities with a small area under cultivation

To account for regional variations in completeness, in our primary analysis communities with a small area under wine cultivation served as reference. Provided that the completeness does not differ systematically between winegrowing communities with a large area under cultivation and adjoining communities with a small area under cultivation, this allows to calculate valid incidence rate ratios by Poisson regression.

Population figures are reported in five year age categories by the State Statistical Office; due to small numbers, the use of a categorized age variable would have caused numerical problems in the regression analysis. Instead, age was included as a continuous variable in the regression analysis (mid-point of each age category). Many factors, like sociodemographic, lifestyle and environmental factors, might considerably differ between large cities and villages/small cities. Cities with more than 100,000 inhabitants (Mainz, Ludwigshafen/Rhein, Koblenz, Kaiserslautern) were therefore excluded from the analysis. Furthermore, we adjusted for rural (<5,000 inhabitants) vs. urban (≥ 5,000 inhabitants) communities. The proportion of community area under fruit cultivation (another potential source of pesticides exposure) was included in the analyses as dichotomous confounder (<5 percent vs. ≥ 5 percent of community area).

All analyses were preformed in SAS [23], stratified by gender and cancer type. The regression analysis includes cancer rate as dependent variable, and age, wine growing area, rural/urban setting and fruit cultivation. All analyses were stratified by gender and diagnosis. The results of our initial Poisson regression indicated a possible problem with overdispersion, which is partly due to heterogeneity between communities with respect to unobserved risk factors. We therefore opted to assume a negative binomial distribution for the dependent variable, which allows to estimate a dispersion parameter *k *for the variance (variance = expected value·(1+*k*·expected value)) and includes the Poisson distribution as a special case (*k *= 0). The negative binomial distribution emerges naturally if expected counts (Poisson parameters) vary among communities according to a gamma distribution. The interpretation of rate ratios stays the same as for Poisson regression. However, results do not substantially differ. For a few rare cancers, the ML fitting algorithm did not converge using the negative binomial distribution. In these cases, estimates from Poisson regression are reported.

### Side analysis of standardized incidence ratios (SIR) using German incidence rates as reference

Even in communities with a small area under cultivation, cancer incidence might be elevated, potentially leading to an underestimation of rate ratios in communities with medium or large area under cultivation. In an additional analysis, we therefore calculated standardized incidence ratios (SIR) regardless of the incompleteness of the Rhineland-Palatinate cancer registry. Standardized cancer incidence ratios were separately computed for winegrowing communities with small, medium, and large area under cultivation using estimated German incidence rates. The expected numbers of cancer (E) for the time period 2000–2003 were compared with the observed numbers (O), calculating standardized incidence ratios (SIR) as the ratio between the observed and expected numbers. Exact 95%-confidence intervals (CI) based on the Poisson distribution of O were calculated.

Results of any analysis based on small numbers are difficult to interpret. Therefore, only those results based on at least ten cases in the respective referent group and ten cases in both comparison groups combined are reported here.

## Results

Tables 2 and 3 present incidence rate ratios (RR) for cancer in males and females for winegrowing communities with medium (> 5 to ≤ 20 percent) and large (>20 percent) area under cultivation compared to communities with small (> 0 to ≤ 5 percent) area under cultivation. Significantly increased RR are observed for non-melanoma skin cancer (C44 ICD-10) among men (RR = 1.32 (95% confidence interval CI 1.20–1.45) for medium and RR = 1.39 (95% CI 1.25–1.54) for a large area under cultivation) as well as among women (RR = 1.40 (95% CI 1.27–1.54) for medium and RR = 1.38 (95% CI 1.23–1.53) for a large area under cultivation).

**Table 2:** Cancer risks (incidence rate ratios RR) in men with residence in communities with a large or medium area under wine cultivation vs. men in communities with low area under wine cultivation

	Reference* (1,665,594 PY^†^)	Area under wine cultivation > 5, ≤ 20% of community area (1,039,435 PY^†^)			Area under wine cultivation > 20% of community area (612,714 PY^†^)		
ICD-10 code	Cases	Cases	RR^‡§^	95% CI	Cases	RR^‡§^	95% CI
Head & neck (C00–C14)	369	188	0.91	0.72–1.15	94	0.86	0.65–1.14
Base of tongue (C01)	35	11	0.53	0.26–1.08	10	0.87	0.40–1.91
Other and unspecified parts of tongue (C02)	35	26	1.22	0.72–2.07	13	1.12	0.56–2.25
Floor of mouth (C04)	42	25	0.98	0.57–1.70	11	0.70	0.34–1.47
Palate (C05)	22	15	1.24	0.62–2.49	5	0.88	0.31–2.54
Other and unspecified parts of mouth (C06)	18	8	0.71	0.29–1.73	3	0.45	0.12–1.71
Parotid gland (C07)	14	8	0.92	0.36–2.30	4	0.80	0.24–2.67
Tonsil (C09)	47	22	0.78	0.45–1.34	13	0.79	0.40–1.56
Oropharynx (C10)	35	15	0.78	0.39–1.57	5	0.48	0.17–1.34
Piriform sinus (C12)	20	10	0.90	0.41–2.02	8	1.67	0.66–4.25
Hypopharynx (C13)	56	27	0.76	0.46–1.26	17	0.88	0.48–1.62
Oesophagus (C15)	156	94	0.96	0.73–1.27	42	0.82	0.57–1.20
Stomach (C16)	241	166	1.06	0.86–1.31	92	1.03	0.79–1.34
Small intestine (C17)	24	14	0.92	0.45–1.86	2	0.21	0.05–0.95
Colon, sigmoid & rectum (C18–C21)	1188	806	1.07	0.95–1.21	460	1.10	0.96–1.26
Colon (C18)	723	473	1.04	0.91–1.20	268	1.06	0.90–1.25
Rectosigmoid junction (C19)	51	42	1.33	0.89–2.00	30	1.68	1.04–2.71
Rectum (C20)	397	284	1.13	0.94–1.35	157	1.10	0.89–1.37
Anus and anal canal (C21)	17	7	0.66	0.26–1.64	5	0.85	0.29–2.50
Liver and intrahepatic bile ducts (C22)	141	73	0.94	0.68–1.30	37	0.88	0.58–1.32
Gallbladder & biliary tract (C23–C24)	76	33	0.67	0.44–1.02	27	0.95	0.59–1.54
Gallbladder (C23)	17	7	0.58	0.23–1.46	7	0.96	0.36–2.53
Other and unspecified parts of biliary tract (C24)	59	26	0.70	0.43–1.13	20	0.95	0.55–1.65
Pancreas (C25)	162	99	1.03	0.78–1.36	51	0.96	0.68–1.37
Nasal cavity and middle ear (C30–C31)	18	9	0.73	0.32–1.67	4	0.51	0.16–1.62
Larynx (C32)	135	78	0.94	0.68–1.29	39	0.88	0.59–1.31
Trachea, bronus and lung (C33–C34)	1039	530	0.98	0.84–1.14	232	0.77	0.64–0.92
Bronchus and lung (C34)	1036	530	0.98	0.84–1.15	232	0.77	0.64–0.92
Bone and articular cartilage (C40–C41)	11	7	0.88	0.32–2.42	3	0.49	0.13–1.89
Skin, malignant melanoma (C43)	230	188	1.32	1.08–1.60	119	1.50	1.18–1.91
Skin, other malignant neoplasms (C44)	1990	1748	1.32	1.20–1.45	959	1.39	1.25–1.54
Mesothelioma (C45)	19	12	1.09	0.52–2.28	5	0.92	0.32–2.68
Other connective and soft tissue (C49)	32	35	1.65	1.00–2.70	9	0.68	0.31–1.48
Breast (C50)	14	9	1.02	0.43–2.39	3	0.60	0.16–2.25
Penis (C60)	20	15	1.17	0.56–2.44	6	0.71	0.26–1.93
Prostate (C61)	1857	1359	1.26	1.12–1.41	787	1.26	1.11–1.43
Testis (C62)	154	107	1.18	0.88–1.60	77	1.31	0.94–1.83
Urinary tract (C64-C66+C68)	330	206	1.03	0.85–1.26	107	0.96	0.75–1.23
Kidney, except renal pelvis (C64)	269	171	1.06	0.86–1.32	89	1.00	0.76–1.31
Ureter (C66)	30	7	0.35	0.15–0.80	7	0.58	0.24–1.39
Bladder (C67, D09.0, D41.4)	699	470	1.16	1.01–1.34	266	1.31	1.10–1.55
Eye and adnexa (C69)	15	8	1.02	0.43–2.43	3	0.88	0.23–3.37
Meninges (C70)	22	20	1.45	0.78–2.69	7	0.88	0.35–2.18
Brain, CNS, meninges (C70–C72, D32–33, D42–43)	133	90	1.08	0.81–1.44	53	1.04	0.73–1.49
Brain (C71, D33, D43)	109	70	1.02	0.74–1.41	45	1.06	0.72–1.57
Thyroid gland (C73)	33	23	1.11	0.64–1.92	16	1.31	0.68–2.53
Hodgkin's disease (C81)	40	24	0.86	0.51–1.46	12	0.64	0.32–1.27
Follicular NHL (C82)	29	21	1.23	0.67–2.25	19	1.98	1.01–3.85
NHL (C82–C85)	197	130	1.12	0.88–1.42	81	1.29	0.96–1.73
Diffuse NHL (C83)	122	66	0.91	0.66–1.24	31	0.80	0.52–1.23
Peripheral and cutaneous T-cell lymphomas (C84)	10	6	0.94	0.33–2.68	8	2.19	0.75–6.37
Other and unspecified types of NHL (C85)	36	37	1.64	1.03–2.59	23	1.84	1.05–3.23
Multiple myeloma (C90)	63	36	0.93	0.60–1.44	21	0.92	0.53–1.59
Leukaemia (C91–C95)	204	113	0.89	0.69–1.15	61	0.84	0.61–1.16
Lymphoid leukaemia (C91)	116	56	0.74	0.53–1.05	28	0.65	0.41–1.02
Myeloid leukaemia (C92)	77	52	1.09	0.75–1.59	27	0.99	0.61–1.60
Primary site unspecified	128	83	0.99	0.75–1.30	43	0.88	0.61–1.28
All malignancies (excluding C44)	7761	5024	1.12	1.05–1.19	2765	1.10	1.03–1.18
All malignancies (including C44)	9751	6772	1.15	1.09–1.22	3724	1.16	1.09–1.23

* Winegrowing communities with >0, <= 5% area under wine cultivation

^† ^PY: Person-Years were approximated by population figures: the sum of population at the end of the year in the years under consideration.

^‡ ^adjusted for age, rural or urban environment, and fruit cultivation

^§ ^Poisson distribution of case counts assumed for: C45, C50, C70

**Table 3:** Cancer risks (incidence rate ratios RR) in women with residence in communities with a large or medium area under wine cultivation vs. women in communities with low area under wine cultivation

	Reference* (1,778,184 PY^†^)	Area under wine cultivation > 5, ≤ 20% of community area (1,098,069 PY^†^)			Area under wine cultivation > 20% of community area (634,060 PY^†^)		
ICD-10 code	Cases	Cases	RR^‡§^	95% CI	Cases	RR^‡§^	95% CI
Head & neck (C00–C14)	123	70	1.05	0.74–1.50	41	1.14	0.75–1.74
Other and unspecified parts of tongue (C02)	14	8	1.01	0.41–2.48	6	1.56	0.52–4.64
Oropharynx (C10)	19	6	0.53	0.20–1.39	5	0.81	0.28–2.39
Oesophagus (C15)	38	17	0.89	0.46–1.70	9	0.91	0.40–2.08
Stomach (C16)	197	147	1.27	1.01–1.60	61	1.07	0.79–1.47
Small intestine (C17)	24	17	1.17	0.64–2.15	8	1.04	0.45–2.40
Colon, sigmoid & rectum (C18–C21)	1122	733	1.04	0.92–1.17	346	0.94	0.81–1.09
Colon (C18)	734	509	1.11	0.97–1.28	214	0.90	0.75–1.08
Rectosigmoid junction (C19)	64	32	0.78	0.51–1.21	20	0.86	0.50–1.48
Rectum (C20)	301	180	0.95	0.78–1.17	108	1.06	0.83–1.35
Anus and anal canal (C21)	23	12	0.89	0.44–1.81	4	0.60	0.20–1.83
Liver and intrahepatic bile ducts (C22)	43	32	1.17	0.72–1.90	19	1.20	0.66–2.19
Gallbladder & biliary tract (C23–C24)	79	58	1.13	0.79–1.61	19	0.64	0.38–1.09
Gallbladder (C23)	39	38	1.44	0.89–2.33	10	0.64	0.30–1.34
Other and unspecified parts of biliary tract (C24)	40	20	0.78	0.46–1.34	9	0.63	0.29–1.34
Pancreas (C25)	158	85	0.97	0.72–1.29	40	0.93	0.64–1.37
Larynx (C32)	19	12	0.99	0.47–2.07	4	0.56	0.18–1.75
Trachea, bronus and lung (C33–C34)	342	168	0.99	0.77–1.27	94	1.19	0.88–1.59
Bronchus and lung (C34)	340	167	0.99	0.77–1.27	94	1.19	0.89–1.60
Skin, malignant melanoma (C43)	274	212	1.17	0.96–1.42	109	1.00	0.78–1.28
Skin, other malignant neoplasms (C44)	1710	1620	1.40	1.27–1.54	807	1.38	1.23–1.53
Retroperitoneum and peritoneum (C48)	10	9	1.72	0.65–4.53	4	1.93	0.53–7.02
Other connective and soft tissue (C49)	30	17	0.98	0.54–1.79	9	1.03	0.46–2.32
Breast (C50)	2525	1527	1.08	0.98–1.20	779	1.01	0.90–1.12
Vulva (C51)	63	36	0.98	0.64–1.50	22	1.23	0.72–2.10
Vagina (C52)	18	20	1.80	0.94–3.45	5	0.82	0.29–2.33
Cervix uteri (C53)	162	97	1.03	0.79–1.34	47	0.94	0.66–1.34
Corpus uteri, (C54–C55)	382	244	1.15	0.94–1.41	146	1.20	0.95–1.52
Corpus uteri (C54)	370	232	1.13	0.92–1.39	144	1.22	0.97–1.54
Uterus, part unspecified (C55)	12	12	1.58	0.70–3.59	2	0.46	0.10–2.17
Ovary and other unspecified female genital organs (C56–C57)	297	196	1.09	0.89–1.34	96	0.97	0.75–1.26
Ovary (C56)	284	183	1.07	0.86–1.32	93	0.99	0.76–1.28
Other and unspecified female genital organs (C57)	13	13	1.66	0.76–3.63	3	0.73	0.19–2.72
Urinary tract (C64-C66+C68)	208	136	1.10	0.86–1.40	73	1.04	0.77–1.40
Kidney, except renal pelvis (C64)	166	116	1.14	0.88–1.48	63	1.09	0.79–1.50
Renal pelvis (C65)	24	10	0.71	0.33–1.49	4	0.55	0.18–1.71
Ureter (C66)	16	10	1.02	0.45–2.29	4	0.75	0.23–2.44
Bladder (C67, D09.0, D41.4)	251	158	1.09	0.88–1.34	85	1.19	0.90–1.56
Brain, CNS, meninges (C70–C72, D32–33, D42–43)	167	105	1.11	0.84–1.46	62	1.25	0.89–1.75
Meninges (C70)	57	46	1.33	0.87–2.03	24	1.29	0.75–2.21
Brain (C71, D33, D43)	107	57	0.98	0.68–1.40	37	1.21	0.79–1.86
Thyroid gland (C73)	102	75	1.20	0.86–1.67	32	0.86	0.56–1.34
Hodgkin's disease (C81)	39	20	0.83	0.48–1.44	9	0.64	0.29–1.39
NHL (C82–C85)	220	114	0.93	0.72–1.21	52	0.78	0.56–1.09
Follicular NHL (C82)	50	18	0.58	0.34–1.01	5	0.29	0.11–0.76
Diffuse NHL (C83)	106	69	1.05	0.76–1.45	25	0.73	0.46–1.17
Other and unspecified types of NHL (C85)	56	24	0.71	0.43–1.14	19	1.07	0.61–1.89
Multiple myeloma (C90)	68	30	0.72	0.46–1.13	21	0.88	0.51–1.49
Leukaemia (C91–C95)	135	65	0.80	0.59–1.09	43	0.98	0.68–1.43
Lymphoid leukaemia (C91)	67	33	0.78	0.51–1.18	22	0.90	0.54–1.50
Myeloid leukaemia (C92)	60	32	0.90	0.59–1.37	16	0.92	0.51–1.66
Primary site unspecified	116	78	1.18	0.90–1.56	36	1.13	0.76–1.68
All malignancies (excluding C44)	7258	4508	1.09	1.03–1.17	2293	1.04	0.97–1.11
All malignancies (including C44)	8968	6128	1.14	1.08–1.21	3100	1.10	1.04–1.17

* Winegrowing communities with >0, <= 5% area under wine cultivation

^† ^PY: Person-Years were approximated by population figures: the sum of population at the end of the year in the years under consideration.

^‡ ^adjusted for age, rural or urban environment, and fruit cultivation

^§ ^Poisson distribution of case counts assumed for: C21, C52, C55, C57, C65, C81

Among men, the rate ratios for a large vs. a small area under cultivation are significantly elevated for the following malignancies: malignant melanoma (C43 ICD-10; RR = 1.50; 95% CI 1.18–1.91), prostate cancer (C61 ICD-10: RR = 1.26; 95% CI 1.11–1.43), bladder cancer (C67 ICD-10; RR = 1.31; 95% CI 1.10–1.55), follicular NHL (C82 ICD-10; RR = 1.98; 95% CI 1.01–3.85) and other and unspecified types of NHL (C85 ICD-10; RR = 1.84; 95% CI 1.05–3.23). In contrast, we find significantly decreased rate ratios for follicular NHL among women (RR = 0.29 (95% CI 0.11–0.76) for a large vs. a small area under cultivation).

Furthermore rate ratios are significantly decreased among men for lung cancer (C34 ICD-10; RR = 0.77; 95% CI 0.64–0.92 for a large vs. a small area under cultivation).

Both men and women showed a slightly elevated RR for all malignancies for communities with medium (men: RR = 1.15; 95% CI 1.09–1.22; women: RR = 1.14; 95% CI 1.08–1.21) as well as with a large area under cultivation (men: RR = 1.16; 95% CI 1.09–1.23; women: RR = 1.10; 95% CI 1.04–1.17).

When non-melanotic skin cancer was excluded, among men, risk ratios for all malignancies remained significantly elevated in communities with medium and a large area under cultivation; among women, solely rate ratios in communities with a medium area under cultivation retained significance.

Tables 4 and 5 present standardized incidence ratios (SIR) for cancer in males and females for winegrowing communities with small (>0 to ≤ 5 percent), medium (> 5 to ≤ 20 percent) and large (>20 percent) area under cultivation using estimated incidence of cancer in the national population of Germany as reference. As the incompleteness of the Rhineland-Palatinate cancer registry would tend to result in potentially considerable underestimation, decreased SIR are not mentioned in the following (and should not be interpreted). The standardized incidence ratios of malignant melanoma remains statistically increased in men (SIR for a medium area under cultivation = 1.17 (95% CI 1.01–1.35), SIR for a large area under cultivation = 1.31 (95% CI 1.08–1.56)). Furthermore, the SIR for prostate cancer remains statistically significant: the SIR is 1.18 (95% CI 1.12–1.24) for a medium area under cultivation and 1.25 (95% CI 1.17–1.34) for a large area under cultivation. The increased incidence of testicular cancer in communities with a large area under wine cultivation is of borderline statistical significance (SIR = 1.26; 95% CI 0.99–1.57). Among women, we find an elevated SIR for endometrial cancer in communities with a large area under cultivation (SIR = 1.43; 95% CI 1.20–1.68). Breast cancer incidence is increased in communities with a medium area under cultivation (SIR = 1.07; 95% CI 1.02–1.12), but not in communities with a large area under cultivation (SIR = 0.99; 95% CI 0.92–1.06).

**Table 4:** Cancer risks (standardized incidence ratios SIR) in men with residence in communities with planted winegrowing areas with the estimated incidence of cancer in the national population of Germany as reference

	Area under wine cultivation > 0, ≤ 5% of community area (1,665,594 PY^†^)				Area under wine cultivation > 5, ≤ 20% of community area (1,039,435 PY^†^)				Area under wine cultivation > 20% of community area (612,714 PY^†^)			
ICD-10 code	Observed	Expected	SIR	95% CI	Observed	Expected	SIR	95% CI	Observed	Expected	SIR	95% CI

Head & neck (C00–C14)	369	238.00	1.13	1.01–1.25	188	205.94	0.91	0.79–1.05	94	117.54	0.80	0.65–0.98
Stomach (C16)	241	421.10	0.57	0.50–0.65	166	262.89	0.63	0.54–0.74	92	144.55	0.64	0.51–0.78
Colon, sigmoid & rectum (C18–C21)	1188	1445.33	0.82	0.78–0.87	806	906.16	0.89	0.83–0.95	460	497.93	0.92	0.84–1.01
Trachea, bronchus and lung (C33–C34)	1039	1376.19	0.75	0.71–0.80	530	865.12	0.61	0.56–0.67	232	478.60	0.48	0.42–0.55
Skin, malignant melanoma (C43)	230	257.40	0.89	0.78–1.02	188	160.19	1.17	1.01–1.35	119	91.08	1.31	1.08–1.56
Prostate (C61)	1857	1833.70	1.01	0.97–1.06	1359	1152.26	1.18	1.12–1.24	787	628.58	1.25	1.17–1.34
Testis (C62)	154	164.80	0.93	0.79–1.09	107	101.97	1.05	0.86–1.27	77	61.20	1.26	0.99–1.57
Urinary tract (C64-C66+C68)	330	392.87	0.84	0.75–0.94	206	247.03	0.83	0.72–0.96	107	137.47	0.78	0.64–0.94
Bladder (C67, D09.0, D41.4)	699	718.87	0.97	0.90–1.05	470	451.25	1.04	0.95–1.14	266	246.29	1.08	0.95–1.22
NHL (C82–C85)	197	255.32	0.77	0.67–0.89	130	160.18	0.81	0.68–0.96	81	90.11	0.90	0.71–1.12
Leukaemia (C91–C95)	204	253.60	0.80	0.70–0.92	113	158.75	0.71	0.59–0.86	61	89.49	0.68	0.52–0.88
All malignancies (excluding C44)	7761	8751.69	0.89	0.87–0.91	5024	5493.56	0.91	0.89–0.94	2765	3041.59	0.91	0.88–0.94

^† ^PY: Person-Years were approximated by population figures: the sum of population at the end of the year in the years under consideration.

**Table 5:** Cancer risks (standardized incidence ratios SIR) in women with residence in communities with planted winegrowing areas with the estimated incidence of cancer in the national population of Germany as reference

	Area under wine cultivation > 0, ≤ 5% of community area (1,665,594 PY^†^)				Area under wine cultivation > 5, ≤ 20% of community area (1,039,435 PY^†^)				Area under wine cultivation > 20% of community area (612,714 PY^†^)			
ICD-10 code	Observed	Expected	SIR	95% CI	Observed	Expected	SIR	95% CI	Observed	Expected	SIR	95% CI

Head & neck (C00–C14)	123	93.17	1.32	1.10–1.58	70	57.01	1.23	0.96–1.55	41	31.13	1.32	0.95–1.79
Stomach (C16)	197	302.45	0.65	0.56–0.75	147	183.37	0.80	0.68–0.94	61	96.98	0.63	0.48–0.81
Colon, sigmoid & rectum (C18–C21)	1122	1529.06	0.73	0.69–0.78	733	929.83	0.79	0.73–0.85	346	491.03	0.70	0.63–0.78
Trachea, bronchus and lung (C33–C34)	342	429.49	0.80	0.71–0.89	168	263.51	0.64	0.54–0.74	94	142.96	0.66	0.53–0.80
Skin, malignant melanoma (C43)	274	314.29	0.87	0.77–0.98	212	191.68	1.11	0.96–1.27	109	107.56	1.01	0.83–1.22
Breast (C50)	2525	2332.19	1.08	1.04–1.13	1527	1429.33	1.07	1.02–1.12	779	786.82	0.99	0.92–1.06
Cervix uteri (C53)	162	239.01	0.68	0.58–0.79	97	145.48	0.67	0.54–0.81	47	83.17	0.57	0.42–0.75
Corpus uteri, (C54–C55)	382	309.64	1.23	1.11–1.36	244	190.05	1.28	1.13–1.46	146	102.37	1.43	1.20–1.68
Ovary and other unspecified female genital organs (C56–C57)	297	449.08	0.66	0.59–0.74	196	273.91	0.72	0.62–0.82	96	149.12	0.64	0.52–0.79
Urinary tract (C64-C66+C68)	208	261.56	0.80	0.69–0.91	136	160.51	0.85	0.71–1.00	73	86.15	0.85	0.66–1.07
Bladder (C67, D09.0, D41.4)	251	325.54	0.77	0.68–0.87	158	200.30	0.79	0.67–0.92	85	107.82	0.79	0.63–0.97
NHL (C82–C85)	220	286.04	0.77	0.67–0.88	114	175.38	0.65	0.54–0.78	52	95.33	0.55	0.41–0.72
Leukaemia (C91–C95)	135	228.14	0.59	0.50–0.70	65	139.57	0.47	0.36–0.59	43	75.32	0.57	0.41–0.77
All malignancies (excluding C44)	7258	8285.78	0.88	0.86–0.90	4508	5070.41	0.89	0.86–0.92	2293	2740.00	0.84	0.80–0.87

^† ^PY: Person-Years were approximated by population figures: the sum of population at the end of the year in the years under consideration.

## Discussion

In this ecological study, a statistically significant positive association with the extent of viniculture is observed for non-melanoma skin cancer in males and females, prostate cancer, bladder cancer, and non-Hodgkin lymphoma in males, but not in females. Lung cancer risk is significantly reduced in communities with a large area under cultivation. Our main hypothesis that pesticides might play a role for the observed associations will be discussed for specific cancer types in the following.

### Specific tumours

#### Non-melanotic skin cancer

Several studies have shown that the lifetime cumulative sun exposure is responsible for the development of non-melanotic skin cancer (for an overview, see [24,25]). In ecologic studies, squamous cell carcinoma is related more strongly to latitude or measured ultraviolet radiation than is basal cell carcinoma. As more outdoor workers might be occupied in regions with extensive winegrowing, our finding of an increased non-melanotic skin cancer risk in winegrowing communities appears plausible. In fact, in communities with a large area under cultivation, 14.8 percent of male skin cancer patients (C44 ICD-10) with known occupation (as recorded in the cancer registry) had worked as an outdoor worker (farmer, winegrower, gardener, forestry worker or construction worker). In communities with medium and a small area under cultivation, this proportion is 12.2 percent and 7.5 percent, respectively. Comparably, the proportion of outdoor workers among female cancer skin cancer patients (C44 ICD-10) is 7.6 percent, 5.1 percent and 2.6 percent in communities with a large, medium and small area under cultivation, respectively. Previous arsenic exposure has to be considered as an alternative explanation: arsenical pesticides were applied by Moselle wine growers [26] between 1920 and 1942. The clinical signs of arsenic exposure are arsenical keratoses, which may progress to squamous cell carcinoma or basal cell carcinoma [27]. Moreover, arsenic seems to act as a co-carcinogen with ultraviolet radiation [27]. As the latency period of non-melanotic skin cancer is suspected to be very long, an excess in non-melanotic skin cancers might therefore be partly explained by arsenic exposure, however, this explanation appears rather speculative. Moreover, risk estimators for non-melanotic skin cancer do not markedly increase when our analysis is restricted to persons aged 70 or more. The association between sun exposure and melanoma of the skin seems to be more complex: Intermittent sun exposure and sunburn history rather than lifetime cumulative sun exposure plays a role in the aetiology of melanoma of the skin [28,29]. This complex relationship might explain why our study does not reveal a clearly increased melanoma incidence in communities with a large area under wine cultivation. Moreover, adjusting for potential confounders as, for example, leisure time UV exposure, was not possible in this study.

#### Brain cancer

While several epidemiological studies point to an increased brain cancer risk among pesticide exposed persons [13,14], few studies specifically focus on the residential population in winegrowing regions. In their ecological study in the province of Trento, Italy, Ferrari and Lovaste [30] find the highest incidence rates of intracranial tumours in regions of intensive fruit and wine cultivation. However, the authors do not indicate the significance level of their findings. Another ecological study among French agricultural workers reveals a significant association between pesticide exposure in vineyards and brain cancer mortality [31]. The results of our ecological study do not support an increased brain cancer risk of residents in winegrowing regions (RR in the primary analysis for large vs. a small area under cultivation = 1.06 (95% CI 0.72–1.57) among men; RR = 1.21 (95% CI 0.79–1.86) among women).

#### Rectum cancer

Some previous studies point to a potentially elevated rectum cancer risk [32,33], other studies find reduced colorectal cancer risks among farmers [34] or farm residents [35]. Altogether, there is very little evidence to date for a possible relationship between pesticide exposure and rectum cancer. Our finding of an increased cancer incidence of the rectosigmoid junction (but not of rectum cancer in all) among males living in winegrowing communities might be alternatively explained by life-style (e.g. dietary) or medical (participation at screening) factors, by inhomogeneous reporting behavior, or by chance.

#### Non-Hodgkin lymphoma

The increased NHL incidence among male, but not among female inhabitants of communities with a medium or large area under wine cultivation suggests a potential occupational rather than residential aetiology. However, in communities with a medium or a large area under cultivation, only two male NHL patients (=2 percent of male NHL patients with known occupation, missing values 55 percent) and one female NHL patient (=1.3 percent of female NHL patients with known occupation, missing values 44 percent) had worked as wine-growers, making an occupational aetiology improbable.

Our finding of an increased NHL incidence among potentially pesticide-exposed residents of winegrowing communities is in accordance with the literature. However, most previous studies are related to agricultural workers in general, not to winegrowing workers. In a large Italian multicenter case-control study [36], orchard, vineyard, and related tree and shrub workers appeared to be at increased risk for hematolymphopoietic malignancies. The carcinogenic effects of pesticides may be associated with their genotoxicity and immunotoxicity [37-39], increased cell proliferation [40], and association with chromosomal aberrations [41]. Because of the lack of a positive association between potential residential pesticide exposure and NHL in females (actually with a significantly decreased rate ratio for follicular NHL in winegrowing communities with a large area under cultivation), our study does not definitely support the hypothesis of an elevated NHL risk among the residential population in Rhineland-Palatinate winegrowing communities.

#### Bladder cancer

To date, there is inconclusive evidence for a relationship between pesticide exposure and bladder cancer. In a retrospective cohort study among 32,600 employees of a lawn care company, Zahm [42] finds a significantly increased bladder cancer mortality. However, bladder cancer numbers are very small; furthermore, two of the three observed deaths had no direct occupational contact with pesticides. Rusiecki et al. [16] evaluate the cancer incidence in atrazine-exposed pesticide applicators among 53,943 participants in the Agricultural Health Study. In their study, assessing atrazine exposure by lifetime days of exposure, the rate ratio for bladder cancer is non-significantly elevated to 3.06 (95% CI 0.86–10.81). Assessing atrazine exposure by intensity-weighted lifetime days, the rate ratio for bladder cancer decreases to 0.85 (95% CI 0.24–2.94). Viel and Challier [17] analyze the mortality from bladder cancer among French farmers. While the mortality among farmers is non-significantly lowered (standardized mortality ratio = 0.96; 95% CI 0.85–1.08), there is a significant association with exposure to pesticides in vineyards (risk ratio = 1.14; 95% CI 1.07–1.22). According to the authors, these results could explain the French south-north gradient in bladder cancer, as vineyards are mainly located in Southern France.

#### Prostate cancer

Our finding of an increased prostate cancer risk in potentially pesticide-exposed residents of winegrowing communities is in accordance with the literature. In a recently conducted meta-analysis, van Maele-Fabry et al. [15] include 18 epidemiological studies published between 1984 and 2004. The combined rate ratio for all studies is 1.28 (95% CI 1.05–1.58). According to the authors, no specific pesticide or chemical class is responsible for the increased risk; nevertheless, the strongest evidence consists for phenoxy herbicides possibly in relation with dioxin and furan contamination. Van Maele-Fabry [15] point to the lack of fundamental understanding of the basic biology of human prostate cancer: hormones (both androgens and estrogens) would likely play a role in the etiology or promotion of prostate cancer. Therefore, the authors regard it as plausible that chemicals able to modulate steroid sex hormones as agonists, antagonists or as mixed agonist-antagonist might contribute to the development of prostate cancer through hormone-mediated effects. Several pesticides might interfere with sexual hormones through direct action on receptors but also through indirect non-receptorial mechanisms.

### Limitations

We applied an ecologic study design which does not allow a differentiation between residential, occupational, and life-style risk factors for cancer. The chief limitation of ecologic studies is the inability to link exposure with disease in particular individuals. A second major limitation of ecologic studies is the lack of ability to control for the effects of potential confounding factors. Thus, observed risk differences between communities with different area under cultivation may be due not to varying levels of pesticide usage, but rather to the independent effect of other confounding variables on cancer risk. Moreover, our "exposure" categories (small, medium, or large area under cultivation) represent very crude indicators of the individual exposure; the actual individual exposure depends on occupation, place of residence at the time of pesticide spraying, wind direction etc. Furthermore, several tests were performed, introducing a multiple comparison problem (altogether, 270 risk ratios were calculated). In general, our study design should therefore be regarded as exploratory rather than hypothesis testing. Due to small numbers, particularly for cancer cases in communities with a large area under cultivation, the power of the study to detect slight increases in incidence is limited. Many other potential risk factors of occupation and lifestyle from living in agricultural area would need to be discussed to explain the findings, but these would have to be collected in a study using individual information. For instance, data on socioeconomic levels or smoking prevalence were not available on a small scale. The use of 1996 data on agricultural characteristics might be criticised, since a lag time of 4–7 years for cancers occurring 2000–2003 is not plausible. It was not possible to obtain older data, but since the political boundaries did not change and agricultural land use stayed constant, their use seems warranted in the current study.

The completeness of reported cancer cases is still relatively low in Rhineland-Palatinate (about 80 percent for all cancers). Therefore, the calculation of standardized incidence ratios for residents of winegrowing communities in comparison with the population of Rhineland-Palatinate might at least partly reflect a higher completeness rather than truly elevated risks. As a probably more reliable approach of calculating cancer risks, we therefore decided to compare the observed cancer cases in communities with a medium or a large area under cultivation with – as a kind of internal reference – the number of cases reported in communities with a small area under cultivation. While we regard the "internal" comparison of winegrowing communities (communities with a medium or large area versus small are under cultivation) as a more reliable approach than the comparison with the Rhineland-Palatinate population, we nevertheless cannot totally exclude a higher (or lower) completeness in communities with a medium or large area under cultivation than in communities with a small area under cultivation.

### Increased incidence of endocrine-related tumors with the estimated incidence of cancer in the national population of Germany as reference

In our primary analysis, we compared cancer rates in communities with a large resp. medium area under cultivation with cancer rates in communities with a small area under cultivation. However, in fact even in communities with a small area under cultivation, cancer incidence might be elevated, potentially leading to an underestimation of the results of our primary analysis (concerning rate ratios in communities with medium or large area under cultivation). In a side analysis, we therefore calculated standardized incidence ratios regardless of the incompleteness of the Rhineland-Palatinate cancer. Because of the incompleteness of the Rhineland-Palatinate cancer registry, the results of the calculation of standardized incidence ratios (SIR) tend to underestimate the true cancer risks for incompletely recorded cancer subentities; therefore decreased SIR should not be interpreted. If standardized incidence ratios were calculated with the estimated incidence of cancer in the national population of Germany as reference, among men we found an elevated SIR for prostate cancer and testicular cancer in communities with a large area under wine cultivation. Among women, we found an elevated SIR for endometrial cancer and (in communities with a medium area under cultivation, but not in communities with a large area under cultivation) for breast cancer incidence. Altogether, the results of our additional SIR analysis are compatible with a potential carcinogenic role of pesticides in the etiology of endocrine-related tumors of the breast, testis, prostate, and endometrium.

## Conclusion

This ecologic study is the first attempt to examine the relationship between cancer incidence and the area under wine cultivation in Rhineland-Palatinate winegrowing communities. The study results point to a potentially elevated skin cancer risk, bladder cancer risk, and endocrine-related (prostate, testicular, breast, and endometrium) cancer risk of the population in communities with a large area under wine cultivation. Mainly due to the ecologic study design, the problem of multiple testing, and due to the insufficient completeness of the Rhineland-Palatinate cancer registry concerning the considered region, these findings are not conclusive for a causal relationship. There is a need for analytic epidemiologic studies differentiating between environmental and occupational exposures to further clarify the cancer risk associated with pesticide usage in wine cultivation.

## Competing interests

The authors declare that they have no competing interests.

## Authors' contributions

AS conceived the study design, coordinated the study and drafted the manuscript, GPH performed the statistical analysis and participated in the study design and coordination, GH, AK, and IS participated in the design of the study and helped to draft the manuscript, JK participated in the statistical analysis and helped to draft the manuscript, MB participated in the coordination of the study and helped to design the study and draft the manuscript. All authors read and approved the final manuscript.
